# Prediction Model and Experimental Verification of Surface Roughness of Single Crystal Diamond Chemical Mechanical Polishing Based on Archimedes Optimization Algorithm

**DOI:** 10.3390/mi16101121

**Published:** 2025-09-30

**Authors:** Zhaoze Li, Xiaoguang Guo, Guanghui Fan, Yueming Deng, Renke Kang, Xuefei Wang

**Affiliations:** State Key Laboratory of High-performance Precision Manufacturing, Dalian University of Technology, Dalian 116024, China; zzli_0726@163.com (Z.L.); guoxg@dlut.edu.cn (X.G.); fanguanghui@mail.dlut.edu.cn (G.F.); dengym@mail.dlut.edu.cn (Y.D.); kangrk@dlut.edu.cn (R.K.)

**Keywords:** single crystal diamond, chemical mechanical polishing, Archimedes optimization algorithm, roughness prediction model

## Abstract

Chemical mechanical polishing (CMP) is a critical technique for fabricating ultra-smooth and high-quality surfaces of single crystal diamond (SCD), where processing parameters profoundly influence polishing performance. To achieve superior diamond surface finishes, this study first investigates the effects of key process parameters, including oxidant concentration, catalyst type, and abrasive particle size, on surface quality through single-factor experiments. Subsequently, an Archimedes optimization algorithm (AOA)-based prediction model for diamond CMP surface roughness (Sa) is developed and validated experimentally. Results reveal that high-concentration oxidants, fine-particle abrasives, and dual-catalyst polishing systems synergistically enhance surface quality. The AOA-based prediction model demonstrates a root-mean-square error (RMSE) of 0.006 and a correlation coefficient (R) of 0.98 between the predicted and experimental Sa values. Under the conditions of a dual-catalyst type, 35% oxidant concentration, and 500 nm abrasive particle size, the model predicts a surface roughness of 0.128 nm, with an experimental value of 0.125 nm and a relative error of less than 3%. These findings highlight the capability of the model to accurately forecast surface roughness across diverse process parameters, offering a novel predictive framework for precision CMP of SCD.

## 1. Introduction

Diamond, renowned as the “ultimate semiconductor”, exhibits exceptional chemical inertness, thermal conductivity, and optical transparency [1,2,3,4,5]. Its ultrahigh hardness, chemical stability, superior thermal conductivity, and outstanding radiation resistance hold profound promise for applications in advanced semiconductor devices, aerospace components, fusion energy systems, ultra-precision machining tools, and high-frequency sensors [6,7,8,9,10]. However, the stringent operational demands in these fields impose extremely rigorous standards on their surface topography and subsurface integrity. Consequently, achieving efficient and high-quality polishing of diamond has become a critical prerequisite for enabling its reliable performance in complex and harsh application scenarios [11,12,13].

Current polishing techniques for SCD predominantly include mechanical polishing, laser polishing, ion beam polishing, plasma polishing [14,15,16,17], and CMP. Kubota et al. [18] used mechanical polishing technology to process single-crystal diamond; although a surface with a Ra of 0.1–0.3 nm was obtained, there are still problems, such as surface scratches and subsurface damage. Doronin et al. [19] found that mechanical polishing can reduce the surface roughness of single crystal diamond to Ra 0.35 nm within a few minutes, but its final surface accuracy still lags behind that achieved by chemical polishing. Luo et al. [20] used infrared picosecond laser polishing to polish single crystal diamond, significantly reducing surface roughness by 41.7% and achieving Ra of 109 nm, but still inducing defects such as microcracks and excessive graphitization. Zou et al. [21] used a microsecond laser to reduce the depth of PCD from 108 nm to 8 nm, significantly improving efficiency. However, due to thermal damage, the residual micrometre-level roughness did not meet the nanometre-level accuracy requirements. Mi et al. [22] used an ion beam to polish a single-crystal diamond, which can remove 95% of the groove depth within 30 min. Yang et al. [23] found through experiments and simulations that FIB etching of diamond exhibits significant crystal orientation effects. Although the sputtering efficiency is higher, it is more prone to amorphization and graphitization damage. In contrast, CMP has emerged as the leading method for achieving ultra-smooth, high-quality diamond surfaces. By integrating chemical reactivity and mechanical action, CMP enables damage-free polishing with superior control over surface quality, making it indispensable for applications requiring atomic-scale precision [24].

The efficiency of CMP is influenced by multiple factors, including polishing pressure, the relative velocity between the workpiece and the polishing disc, oxidant–catalyst system type, abrasive particle characteristics, and the polishing fluid flow rate [25]. Oxidants are critical for initiating redox reactions, as they react with the diamond surface to form a softened oxide layer, enabling damage-minimized material removal through subsequent mechanical action. Common oxidants used in diamond CMP include NaNO_3_, KNO_3_, KOH, KClO_3_, K_2_Cr_2_O_7_, H_2_O_2_, HClO, HNO_3_, H_2_SO_4_, Cr_2_O_3_, KMnO_4_ and others. Among these, Fenton reaction systems are renowned for their strong oxidizing capability. They have drawn extensive attention due to their ability to significantly promote redox reactions and enhance polishing efficiency [26,27,28,29,30]. Recent studies have demonstrated the superiority of Fenton-based slurries in achieving high-quality diamond surfaces. Shi et al. [31] compared surface roughness under different oxidants and found that Fenton solutions yielded the lowest values for SCD. Guo et al. [32] further optimized the process by adding W0.5 diamond micro powder to Fenton solutions, achieving a high-quality SCD surface with nanoscale roughness and localized atomic-level smoothness. Liao et al. [33] introduced K_2_FeO_4_ into Fenton systems, enabling high-quality CMP of SCD with minimal subsurface damage, as evidenced by ultra-low roughness and thin damage layers. These findings collectively highlight Fenton reactions as a leading technology for precision diamond polishing, integrating chemical reactivity and mechanical action to meet the stringent requirements of advanced semiconductor and optical applications.

Catalysts, particularly metal ions, are pivotal in achieving high-quality SCD surfaces via Fenton-based CMP. These catalysts mediate redox reactions with H_2_O_2_ to generate highly reactive hydroxyl radicals (·OH), the key oxidizing species for diamond surface modification [34]. Considerable research has focused on Fenton-like systems incorporating transition metals such as Cu, Co, Mn, and Ce, with iron (Fe^3+^) and copper (Cu^2+^)-based catalysts emerging as particularly effective candidates [35,36,37]. Luo et al. [38] demonstrated that Fe^3+^-catalyzed Fenton reactions under acidic conditions efficiently decompose H_2_O_2_ to produce ·OH, which oxidizes the SCD surface to form oxygen-containing functional groups. This process enabled significant material removal and ultra-smooth finishes, highlighting the dual role of chemical oxidation and mechanical action in CMP. Chen et al. [39] advanced this concept by developing copper-doped dendritic mesoporous silica nanoparticles, where Cu^2+^ catalyzes heterogeneous Fenton reactions to enhance polishing efficiency and achieve atomic-level planarization of the dielectric films. Wang et al. [40] further expanded the applicability of Fenton-like systems by designing Fe/Cu composite catalysts, which synergistically degrade organic pollutants across a broad pH range (3–9) through dual-metal activation of H_2_O_2_. This innovation overcomes the strict pH limitation of the traditional Fenton process, demonstrating the multi-metal of multi-metal catalyst systems.

While substantial research exists on surface roughness prediction models, the specific challenge of modelling diamond CMP remains underdeveloped. Current approaches primarily rely on mathematical theories to correlate experimental parameters such as abrasive properties, oxidant characteristics, pH, and polishing pressure, Sa, and material removal rate (MRR) [41]. There is an urgent need to conduct research on new predictive models. In recent years, significant progress has been made in intelligent algorithms, which have shown broad application prospects in optimization scheduling, power prediction, and parameter optimization [42]. However, existing algorithms struggle to achieve an ideal balance between solution efficiency and result accuracy when dealing with complex optimization problems, and the adaptability of algorithm performance varies significantly in different application scenarios. The AOA, constructed based on mathematical principles, provides a new solution for this field. This algorithm exhibits unique advantages in convergence speed and global optimization ability when facing high-dimensional complex function optimization tasks by introducing multiple sets of adaptive parameter adjustment mechanisms [43]. Although the AOA has been widely applied in parameter optimization, its application in the CMP of SCD remains limited. This algorithm has a huge data processing system and rigorous theoretical framework, which is expected to break through the limitations of traditional empirical modelling and provide new ideas for optimizing CMP process parameters [44].

This study bridges this gap by preliminarily analyzing the impact of catalyst type, oxidant concentration, and abrasive particle size on diamond surface roughness via single-factor experiments. Leveraging the AOA, we develop and validate a predictive model, achieving a prediction error below 3%. The results establish a novel framework for optimizing CMP parameters, combining mechanistic insights with advanced algorithms to enhance polishing quality and efficiency for diamond applications in semiconductors and precision engineering.

## 2. Preliminary Analysis of Process Parameters on Polishing Effect

Luo et al. [38] found that oxidant concentration has a significant impact on the polishing performance of SCD. Huang et al. [45] found that grinding particle size has a significant impact on the polishing performance of SCD. Yang et al. [46] found that the Catalyst type has a significant impact on the polishing performance of SCD. Therefore, the experimental plan aims to investigate the effects of three factors, including oxidant concentration, abrasive particle size, and catalyst type, on the polishing effect.

**Figure 1:**
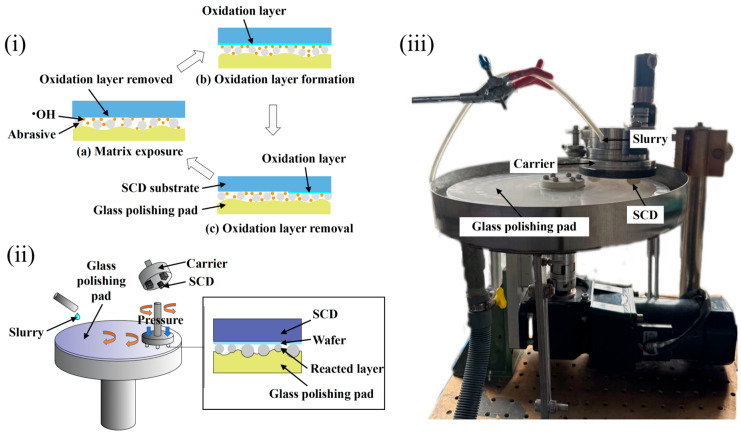
Material removal principle, working principle, and CMP equipment constructed. (**i**) Material removal principle; (**ii**) working principle; (**iii**) CMP equipment constructed.

Firstly, the research plan aims to conduct preliminary experiments on the three variables mentioned above. The material removal principle, working principle, and CMP equipment setup are shown in Figure 1. During polishing, the slurry flowed over the polishing pad and infiltrated the interface between the diamond wafer and the polishing pad. The oxidant initiated a chemical reaction with the diamond surface, forming a softened oxide layer on the surface. Under the combined action of mechanical forces (from the rotating pad, abrasive particles, and wafer) and the chemical reactivity of the slurry, this oxide layer was efficiently removed, yielding a high-quality polished surface.

**Figure 2:**
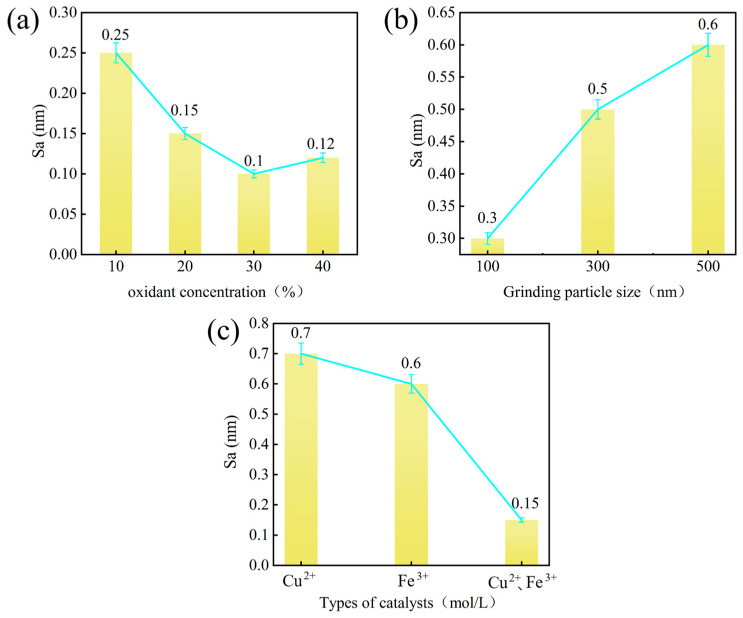
The influence of different parameters on surface roughness. (**a**) oxidant concentration; (**b**) abrasive particle size; (**c**) catalyst type.

Figure 2 shows the effects of these parameters on surface roughness: Figure 2a shows the impact of oxidant concentration. As the concentration increases, SCD surface roughness initially decreases and then increases, with the lowest roughness achieved at 30% oxidant concentration. This trend reflects the balance between efficient oxidation layer formation at moderate concentrations and excessive chemical etching at higher levels. Figure 2b shows the effect of abrasive particle size. Surface roughness slightly increases with larger particle sizes, though the effect remains minimal, indicating that mechanical action is not significantly reflected in surface roughness. Figure 2c shows the influence of catalyst type. Dual-catalyst systems (Fe^3+^/Cu^2+^) consistently yield lower surface roughness than single catalyst counterparts, demonstrating synergistic enhancement in ·OH generation and diamond surface oxidation. Further mechanistic analysis reveals that the combined action of Fe^3+^ and Cu^2+^ in the polishing solution accelerates Fenton-type reactions, promoting the formation of oxygen-containing functional groups on the diamond surface. This dual-catalyst synergy enhances the efficiency of oxide layer removal during CMP, thereby achieving a high-quality surface.

The Cu^3+^/Cu^2+^ and Fe^3+^/Fe^2+^ polishing solution redox cycles exhibit remarkable synergistic catalytic effects. The Cu^3+^/Cu^2+^ cycle that characterized by a significantly higher electrode potential than Cu^2+^/Cu^+^, which provides the robust thermodynamic driving force for H_2_O_2_ activation. This high potential facilitates electron transfer, enabling Cu^3+^ to efficiently accept electrons and reduce to Cu^2+^ while promoting subsequent H_2_O_2_ decomposition. Driven by this cycle, H_2_O_2_ generates highly reactive ·OH. Concurrently, the Fe^3+^/Fe^2+^ cycle complements the Cu^3+^/Cu^2+^ pathway, jointly accelerating ·OH generation. This synergistic mechanism enhances catalytic efficiency and demonstrates unique advantages in sustaining H_2_O_2_ decomposition for radical production. The underlying reactions are shown in Equations (1)–(8).

(1) $${\mathrm{Cu}}^{2+}+H_{2}O_{2}\to {\mathrm{Cu}}^{3+}+\cdot \mathrm{OH}+{\mathrm{OH}}^{-}$$

(2) $${\mathrm{Fe}}^{3+}+H_{2}O_{2}\to {\mathrm{HO}}_{2}\cdot +\;{\mathrm{Fe}}^{2+}+H^{+}$$

(3) $${\mathrm{Cu}}^{3+}+H_{2}O_{2}\to {\mathrm{Cu}}^{2+}+{\mathrm{HO}}_{2}\cdot +H^{+}$$

(4) $${\mathrm{Cu}}^{3+}+{\mathrm{Fe}}^{2+}\to {\mathrm{Cu}}^{2+}+{\mathrm{Fe}}^{3+}$$

(5) $${\mathrm{Fe}}^{2+}+H_{2}O_{2}\to {\mathrm{Fe}}^{3+}+\cdot \mathrm{OH}+{\mathrm{OH}}^{-}$$

(6) $${\mathrm{Cu}}^{2+}+H_{2}O_{2}\to {\mathrm{Cu}}^{+}+{\mathrm{HO}}_{2}\cdot +\;H^{+}$$

(7) $${\mathrm{Cu}}^{+}+H_{2}O_{2}\to {\mathrm{Cu}}^{2+}+{\mathrm{HO}}_{2}\cdot +\;{\mathrm{OH}}^{-}$$

(8) $${\mathrm{HO}}_{2}\cdot +\;H_{2}O_{2}\to O_{2}+H_{2}O+\cdot \mathrm{OH}$$

**Figure 3:**
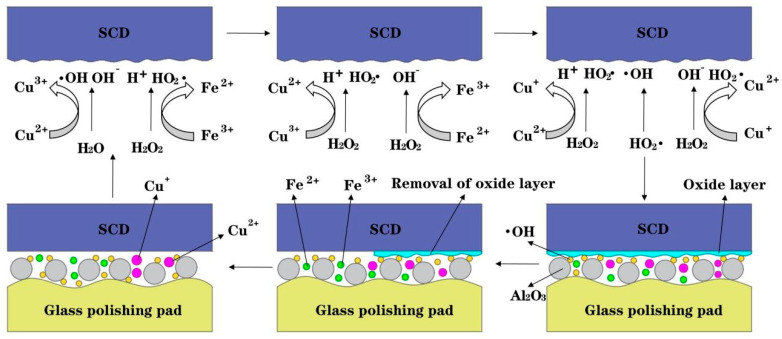
Schematic diagram of Fe^3+^ and Cu^2+^ promoting H_2_O_2_ decomposition.

The mechanism by which H_2_O_2_ decomposition is promoted by Fe^2+^ and Cu^+^ is shown in Figure 3. As electron mediators, reduced metal ions (Fe^2+^, Cu^+^) significantly lower the activation energy of H_2_O_2_ decomposition via Fenton-type reactions, generating highly oxidative ·OH. When these metal ions form coordination complexes with surface hydroxyl groups, electron transfer from ligand orbitals to metal centres occurs, yielding highly reactive excited-state intermediates. These intermediates induce O-O bond cleavage through intramolecular electron rearrangement, coupled with proton-electron transfer, to produce ·OH and regenerate catalytic active sites. This multi-step electron transfer mechanism expands the spatiotemporal scope of catalysis and enhances overall reaction efficiency by optimizing quantum yield.

## 3. Single Factor Experiments

To further investigate the effects of oxidant concentration, abrasive particle size, and catalyst type on the polishing performance, constant variables were maintained throughout the experiments, including polishing disc speed, workpiece rotation speed, applied pressure, and abrasive concentration. As shown in Table 1, the CMP process parameters for SCD were defined as follows:

The preparation of the CMP solution was critical to the experiment. Considering the combined effects of mechanical factors (abrasive particle size) and chemical factors (catalyst type and oxidant concentration), CMP experiments on SCD were performed using phosphoric acid (H_3_PO_4_) as a pH regulator, with a uniform pH setting of 3.2, alumina (Al_2_O_3_) as abrasive, deionized water as a solvent, and varying oxidant types. Sixteen polishing solutions (labelled SL1–SL16) with specific compositions were prepared, as shown in Table 2, to ensure experimental comprehensiveness. A single-variable control method was employed to analyze the impact of each factor. Specifically, oxidant concentration was varied in SL1–SL8 and SL9–SL16. At the same time, other parameters were held constant, allowing for the isolation of its influence on surface roughness. Catalyst type: In SL3, SL4, SL7, SL5, SL6, and SL8, the catalyst type was altered while maintaining other conditions, facilitating analysis of its influence on surface roughness. Abrasive particle size: In SL3, SL5, SL4, and SL6, abrasive particle size was adjusted as the sole variable to evaluate its influence on surface roughness.

## 4. Results and Discussion

Different combinations of oxidant concentration and abrasive particle size yield distinct outcomes across catalyst systems. To dissect the role of individual variables within this complex interplay, this study employed a preliminary approach integrating experimental design and multi-dimensional result analysis. The polished surface morphology, contour, and roughness were characterized using a 3D surface optical profilometer (NewView 9000, ZYGO, Middlefield, OH, USA), with measurements acquired over an area of 868 × 868 μm^2^. Sa values represent the average of six independent measurements, ensuring statistical reliability.

### 4.1. The Influence of Catalyst Types on Surface Roughness and Surface Quality

**Figure 4:**
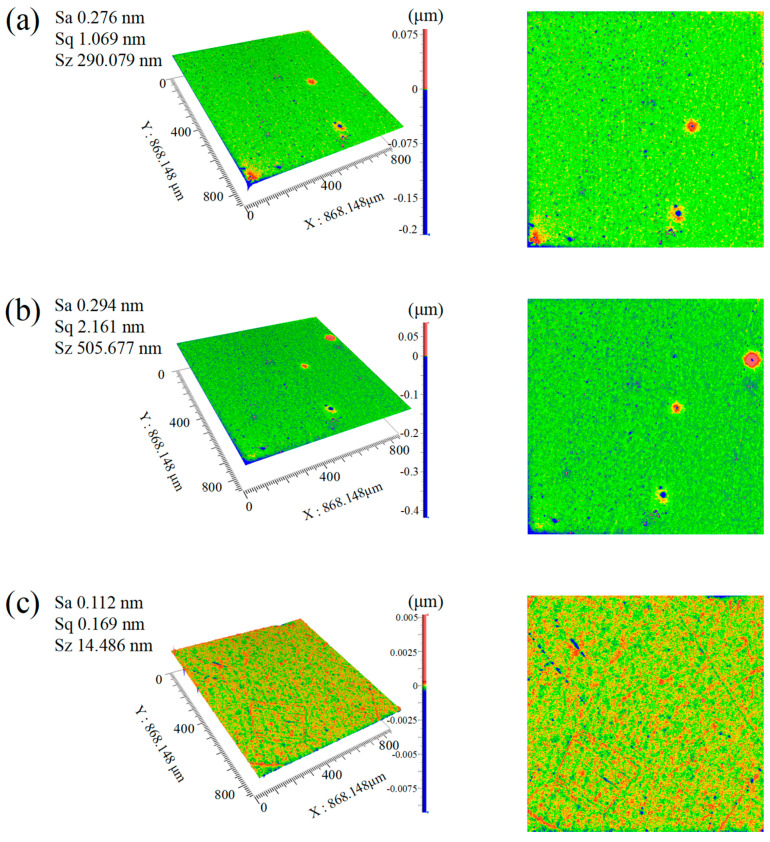
Surface morphology captured by Zygo in 3D (**left** image) and 2D (**right** image). (**a**) SL5; (**b**) SL6; (**c**) SL8.

In experiments comparing polishing solutions SL5, SL6, and SL8, the synergistic design of the catalyst system was found to significantly influence the surface quality of SCD in CMP. The study preliminarily regulated catalyst types: single catalyst systems (Fe_2_(SO_4_)_3_ in SL5 and CuSO_4_ in SL6) and a dual-catalyst system (Fe_2_(SO_4_)_3_ + CuSO_4_ in SL8). As shown in Figure 4, the polishing effects varied markedly: under the dual-catalyst system, the Sa of SCD reached 0.112 nm, compared to 0.276 nm for Fe_2_(SO_4_)_3_ and 0.294 nm for CuSO_4_ alone. These results demonstrate that the dual-catalyst system enhances catalytic efficiency by approximately 60% relative to single-catalyst systems, significantly improving surface quality.

### 4.2. Effect of Oxidant Concentration on Surface Roughness

**Figure 5:**
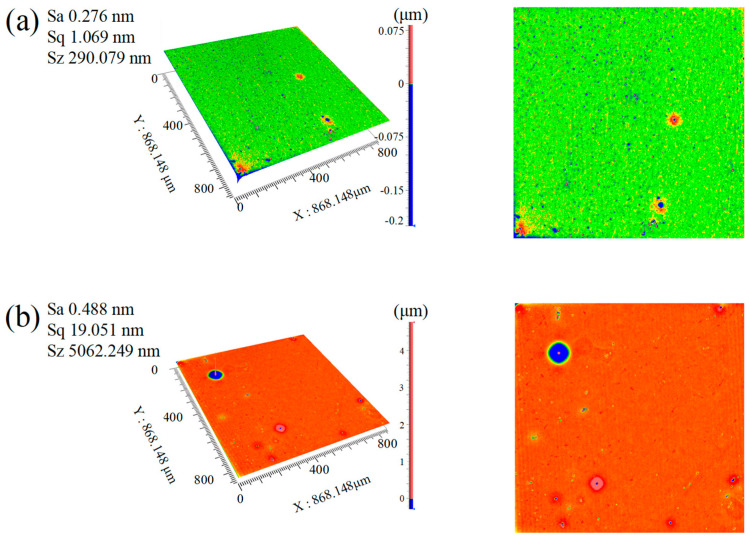
Comparison of 3D (**left** image) and 2D (**right** image) surface morphology contours captured by Zygo. (**a**) SL5; (**b**) SL13.

To investigate the effect of oxidant concentration on surface roughness, an experimental plan was designed to compare SL5 (30% oxidant concentration) and SL13 (15% oxidant concentration). With abrasive particle size and all other process parameters strictly controlled identically, only the oxidant concentration was varied to isolate its impact on the polishing effect. In-depth analysis revealed significant differences in surface roughness values between the two groups, as illustrated in the experimental results shown in Figure 5.

The experimental results demonstrate a pronounced effect of oxidant concentration on the surface quality of SCD. As the hydrogen peroxide concentration increased from a low concentration (SL13) to a high concentration (SL5), the Sa significantly decreased from 0.489 nm to 0.276 nm, a reduction of approximately 43%. This result highlights the critical role of oxidant concentration in optimizing surface smoothness during CMP.

### 4.3. The Influence of Abrasive Particle Size on Surface Quality

**Figure 6:**
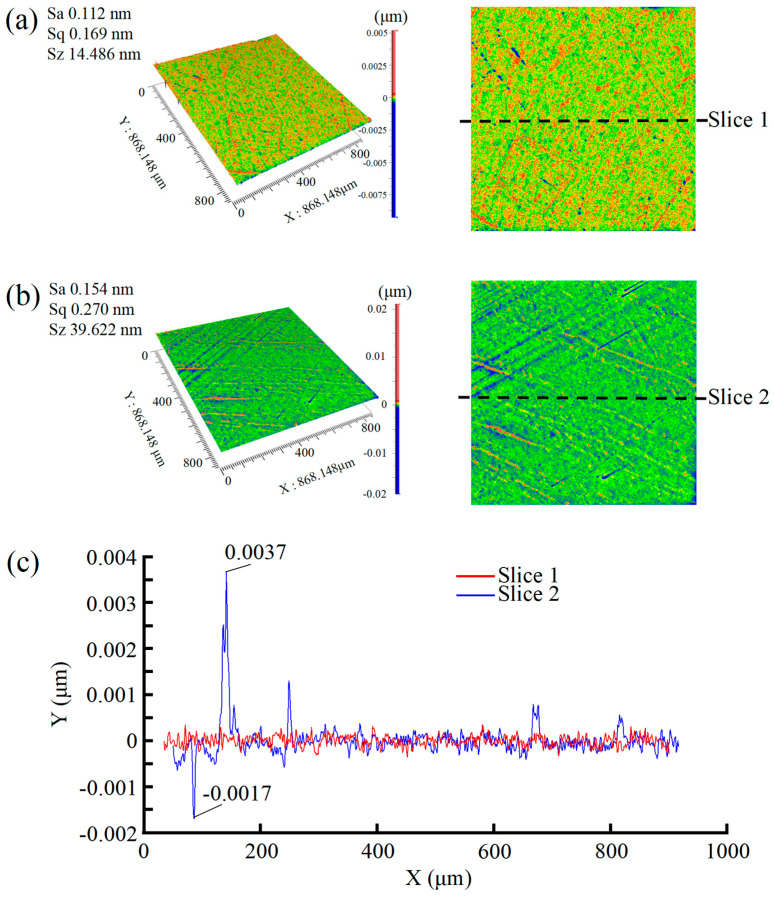
Comparison of 3D (**left** image) and 2D (**right** image) surface morphology contours captured by Zygo. (**a**) SL8; (**b**) SL7; (**c**) cross-sectional surface.

A comparative analysis was performed on two samples, SL7 (500 nm abrasive) and SL8 (100 nm abrasive), with catalyst type and all other process parameters strictly controlled. Distinct surface morphology differences were observed by adjusting only the abrasive particle size. As shown in Figure 6, while the Sa of the two groups were comparable, their surface quality varied significantly: The 500 nm abrasive system achieved nanoscale roughness (Sa ≈ 0.15 nm) but exhibited a dense network of periodic scratches and surface protrusions. High-resolution characterization revealed microdamage with scratch depths of 2 nm and protrusion heights of 4 nm. Reducing the abrasive size to 100 nm significantly improved surface quality. Although Sa decreased slightly to 0.11 nm, scratch density and depth were reduced by an order of magnitude compared to the 500 nm system. These results demonstrate that smaller abrasive particles offer distinct advantages in minimizing surface microdamage, leading to smoother and more integral SCD surfaces. The reduction in abrasive size effectively mitigates mechanical scratches and enhances surface integrity, even with marginal changes in roughness values.

**Figure 7:**
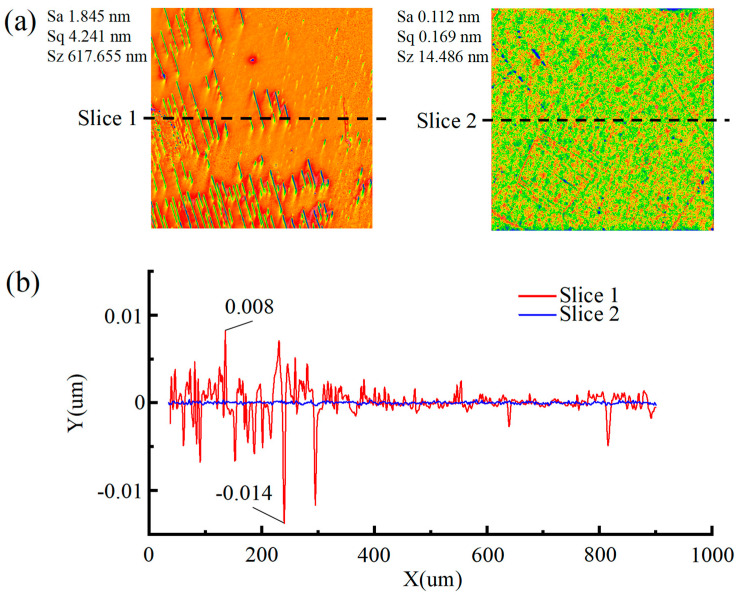
Comparison of surface morphology contours captured by Zygo. (**a**) SL9; (**b**) cross-sectional surface.

Experimental results, as depicted in Figure 7, indicate that optimal polishing performance for SCD is achieved by using a small abrasive particle size, high concentration oxidant, and a dual-catalyst system. This combination yields a Sa of only 0.112 nm, accompanied by excellent surface quality. In stark contrast, the SL9 polishing solution, which uses a large abrasive particle size, low oxidant concentration, and no catalyst, produces significantly inferior results. Post-polishing, the surface exhibits a high Sa of 1.8 nm, characterized by a dense network of periodic scratches and prominent peaks. Microscopic analysis reveals that the scratch depth reaches up to 14 nm, while the peak height measures 8 nm.

## 5. Diamond CMP Surface Roughness Prediction Model Based on AOA

### 5.1. Algorithm Introduction

The AOA is inspired by the principle of fluid statics, as described by Archimedes, which is a swarm intelligence optimization method. Its fundamental concept revolves around mimicking the adaptive motion of objects submerged in viscous fluids. During initialization, the AOA randomly generates a population of candidate solutions within the solution space, evaluating the quality of each individual by using a fitness function. In the iterative optimization phase, individuals adjust their ‘physical attributes’ by utilizing the difference between their current health status and the best experience of the group, which is a metaphorical representation of solution parameters. This adjustment modulates their motion characteristics in the virtual fluid environment, mirroring the dynamic interplay between buoyancy and resistance forces. As individuals update their positions according to fluid-influenced rules, they execute quasi-Brownian motion, transitioning from random exploration to targeted search. This iterative process gradually guides the population towards the vicinity of the global optimal solution. The definitions of relevant parameters are shown in Table 3.

### 5.2. Roughness Prediction Model Based on AOA

In this study, the key influencing factors of surface roughness, including catalyst type, abrasive particle size, and oxidant concentration, are integrated into the AOA to develop a surface roughness prediction model. The algorithm achieves this by iteratively simulating these three variables as dynamic parameters within its optimization framework.

According to Equations (9)–(13), the algorithm generates a random initial population, where a uniform distribution defines the position of each individual in the search space. Concurrently, three critical parameters, including density (*ρ*), volume (*V*), and resistance coefficient (*k*), are randomly initialized within the (0,1) interval for each individual. These parameters metaphorically represent the relative influence weights of catalyst type, abrasive particle size, and oxidant concentration, respectively, establishing a mapping between physical properties in the AOA framework and the actual process variables of the polishing system. This initialization strategy ensures that the algorithm can efficiently explore the multi-dimensional parameter space from the outset.

(9) $$X_{i}^{0}=X_{\min}+rand(0,1)\cdot (X_{\max}-X_{\min})$$

(10) $$\rho_{i}^{0}=rand(0,1)$$

(11) $$\;V_{i}^{0}=rand(0,1)\;$$

(12) $$a_{i}^{0}=\frac{\rho_{i}^{0}}{V_{i}^{0}+\epsilon }$$

(13) $$\;k_{i}^{0}=k_{\min}+rand(0,1)\cdot (k_{\max}-k_{\min})$$

The initial fitness of each individual is jointly determined by these parameters, with the optimal individual selected through fitness evaluation to record its position, density (*ρ*), resistance coefficient (*k*), and volume (*V*). Parameters are constrained within feasible ranges (e.g., analogous to neural network weights typically bounded in [−1, 1]). The neural network architecture defines the dimensionality of each solution: with three nodes in the input layer (oxidant concentration, abrasive particle size, catalyst type), the hidden layer has n nodes, and the output layer has one roughness prediction node. This framework ensures that the movement of each individual in the solution space iteratively optimizes the correlation between input parameters and predicted surface roughness, guided by the AOA adaptive adjustment mechanism.

Individual density (*ρ*), a key parameter for determining position updates, is calculated using the formula shown in Equation (14).

(14) $$\rho_{i}^{t+1}=\rho_{i}^{t}+\alpha \cdot (\rho_{best}-\rho_{i}^{t})+\beta \cdot rand(\;)\;$$

This formula updates the density value of each individual, which algorithmically corresponds to the catalyst type. The new density is computed through a tripartite mechanism: (1) The difference between the current individual density and the optimal population density α·(*ρ_best_* − *ρ_i_^t^*), (2) a stochastic perturbation term introducing exploratory variance β·*rand*( ), and (3) the prior density value *ρ_i_^t^* to ensure temporal continuity.

Analogously, the key determining factor for updating individual volume position is shown in Equation (15).

(15) $$V_{i}^{t+1}=V_{i}^{t}+\gamma \cdot (V_{best}-V_{i}^{t})+\delta \cdot rand(\;)$$

This formula updates the volume value of each individual, which is mapped to the adjustment of abrasive particle size within the algorithm. The update mechanism parallels that of the density formula, consisting primarily of three components: (1) the difference between the current volume and the optimal volume within the population γ·(*V_best_* − *V_i_^t^*), (2) random perturbation terms that introduce variability for exploration δ·*rand*( ) and (3) the historical volume values of the individual to maintain continuity *V_i_^t^*.

Following this, the calculation formula for the drag coefficient is Equation (16).

(16) $$k_{i}^{t+1}=k_{i}^{t}+\varphi \cdot (k_{best}-k_{i}^{t})+\omega \cdot rand(\;)-k_{i}^{t}\cdot sign(k_{i}^{t}-k_{avg})$$

This formula simulates the impact of oxidant concentration on polishing quality by modelling the resistance coefficient’s influence on individual position updates, with its calculation incorporating four components: (1) the difference between the current resistance coefficient and the optimal coefficient φ·(*k_best_ − k_i_^t^*), (2) a stochastic random perturbation term for solution space exploration ω·*rand*( ), (3) direct adjustment of deviating individuals to guide them towards the optimal trajectory *k_i_^t^*·*sign*(*k_i_^t^ − k_avg_*) and (4) the historical value of the resistance coefficient to maintain evolutionary continuity *k_i_^t^*.

As shown in Equation (17), based on the updated density, volume, and resistance coefficients obtained through the above mechanisms, the algorithm then calculates the normalized acceleration of each individual.

(17) $$Acc_{i}^{t}=\frac{\rho_{i}^{t}+V_{i}^{t}}{\rho_{best}+V_{best}+\varepsilon }\cdot rand\cdot (X_{best}-X_{i}^{t})+k_{i}^{t}\cdot \mathrm{sign}(Acc_{i}^{t}-Acc_{avg})$$

The ratio within the formula (*ρ_i_^t^ + V_i_^t^*)/(*ρ_best_ + V_best_+ε*) serves as a critical regulatory factor: when the parameter of the individual approaches the optimal value, the ratio tends toward 1. Acceleration is primarily directionally driven by (*X_best_* − *X_i_^t^*), guiding the individual to converge toward the current optimal region (local exploitation). Conversely, if an individual deviates from the optimal value, the ratio decreases. Amplifying the influence of the random term *rand*( ) to prompt the individual to explore new regions (global exploration). Among them, a minimal positive number (*ε*) is incorporated to avoid denominator-zero errors.

According to the individual acceleration, the formula that the algorithm updates the position of the individual is shown in Equation (18).

(18) $$X_{i}^{t+1}=X_{i}^{t}+C_{1}\cdot Acc_{i}^{t}+C_{2}\cdot rand(0,1)\cdot (X_{\mathrm{best}}^{t}-X_{i}^{t})$$

C_1_ (typically set to 1) is used to regulate acceleration and guide the search direction. C_2_ (typically set to 2) is used to amplify exploration during early iterations when volumes are significant, prevent premature convergence, and preserve population diversity. After updating positions, the algorithm recomputes the fitness of all individuals and updates the global optimal parameters.

The fitness quantifying the discrepancy between the predicted roughness (derived from the model) and the actual measured roughness is shown in Equation (19).

(19) $$Fitness_{i}=\frac{1}{N}\sum_{k=1}^{N}{\left(y_{\mathrm{pred},k}-y_{\mathrm{true},k}\right)}^{2}$$

Here, *N* is the number of training samples, predicted value *y_pred,k_* is calculated from the current parameters and input data $X_{i}^{t}$. Updated parameters *X_i_^t+^*^1^ are reintroduced into the neural network for forward propagation to generate predicted values and compute fitness. The algorithm iterates until the volume parameter decays to near zero or the prediction error converges to a preset threshold. The iteration count is set to 30, and MATLAB 2023b is employed as the simulation software to execute the model.

### 5.3. Testing and Analysis

In the AOA, the training set, validation set, and test set serve as critical indicators for evaluating algorithm performance. The training set adjusts model parameters through continuous iteration to minimize prediction errors. The validation set monitors model performance in real-time to prevent overfitting and ensure good generalization ability. The test set evaluates the performance of the model on unseen data to validate the final performance of the optimized model.

**Figure 8:**
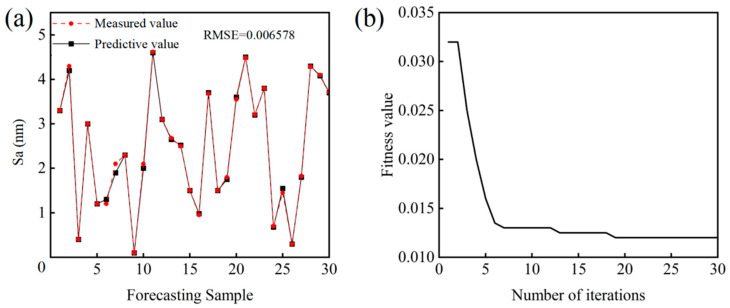
Relative error curve and iteration curve between measured and predicted values. (**a**) Relative error curve; (**b**) Iteration curve.

As shown in Figure 8a, the root mean square error (RMSE) of polished surface roughness is only 0.06578, demonstrating the high effectiveness of the established prediction model in accurately capturing the relationship between the three variables (oxidant concentration, abrasive particle size, and catalyst type) and polished Sa to enable precise surface roughness prediction. The convergence process of the model is shown in Figure 8b. The fitness value decreases significantly during the initial training phase, reflecting continuous learning and optimization of the model. The curve gradually stabilizes as the number of iterations reaches 10–15, indicating gradual convergence. Finally, the fitness value plateaus at 20 iterations, signifying that the model has achieved the required accuracy. These results show that the surface roughness prediction model achieves rapid convergence and ensures an efficient prediction process, demonstrating high efficiency and reliability in practical applications.

**Figure 9:**
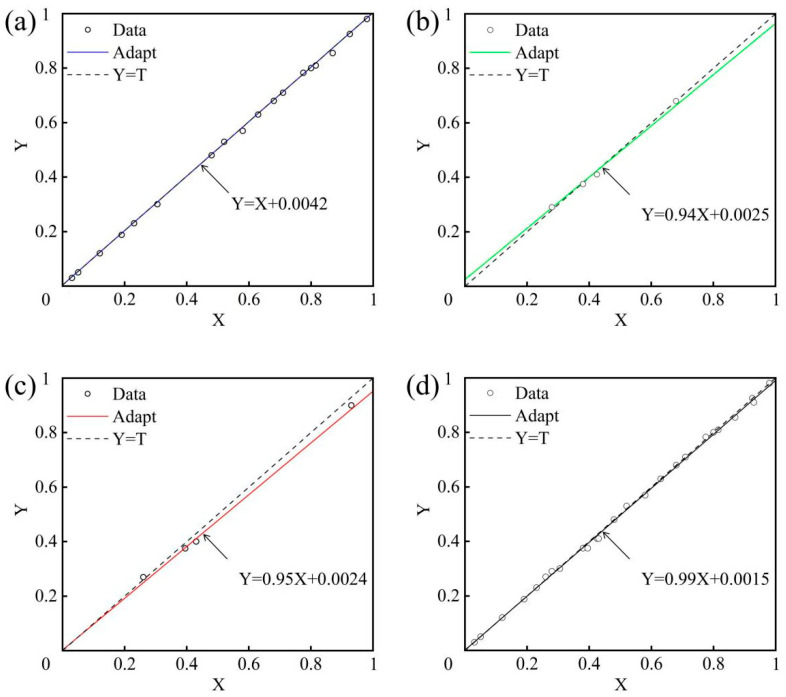
Regression curve analysis. (**a**) Training set; (**b**) Validation set; (**c**) Test set; (**d**) Aggregate set.

To verify the accuracy of the surface roughness prediction model, regression equations were employed for in-depth analysis, with the correlation coefficient (R) serving as a quantitative metric. The experimental results are shown in Figure 9a–d. The correlation coefficients R for the training set, validation set, testing set, and combined dataset are 1, 0.94, 0.95, and 0.99, respectively. This outcome strongly indicates a high linear correlation between predicted and measured values. A detailed examination of the correlation coefficient R was conducted to assess the further performance of the model using the AOA. Among them, the training set exhibits an R close to 1, demonstrating precise fitting to the training data and its ability to predict surface roughness trends accurately. The validation and test sets, with the R of 0.94 and 0.95, respectively, underscore the prediction accuracy of the model on independent samples, indicating its robust generalization capability. Finally, the R of the combined dataset is 0.99, which reflects the overall stability across diverse data scenarios of the model, confirming its reliability and effectiveness in practical applications.

### 5.4. Experimental Result Verification

**Figure 10:**
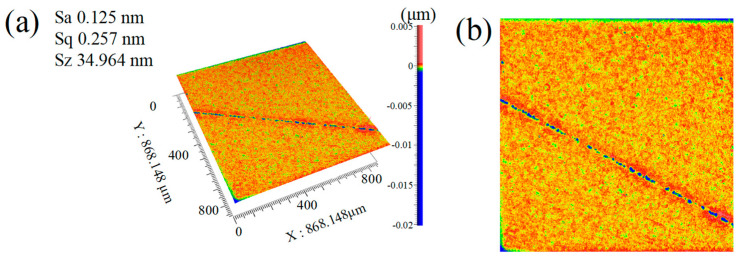
Surface morphology of S4 captured by Zygo. (**a**) 3D (**left** image); (**b**) 2D (**right** image).

To validate the accuracy of the surface roughness prediction model developed using the AOA, this study employed four additional experimental datasets (S1–S4) for model verification. The specific process parameters for each dataset are as follows: S1: 10% oxidant concentration, 500 nm abrasive particle size, single Cu^2+^ catalyst. S2: 5% oxidant concentration, 500 nm abrasive particle size, no catalyst. S3: 30% oxidant concentration, 300 nm abrasive particle size, single Fe^3+^ catalyst. S4: 35% oxidant concentration, 500 nm abrasive particle size, Fe^3+^-Cu^2+^ dual catalysts shown in Table 4, comparative analysis reveals excellent agreement between the predicted values and actual measured values of the model, with relative errors consistently below 3%. For instance, the experimental morphology of sample S4, as shown in Figure 10, when the oxidant concentration is 35%, the abrasive particle size is 500 nm, and Fe^3+^ and Cu^2+^ dual catalyst is used, the measured value of Sa is 0.125 nm, and the predicted value of Sa in the model is 0.128 nm.

In conclusion, the prediction model demonstrates the reliable capability to predict the Sa of SCD within the established error tolerance.

## 6. Conclusions

This study preliminarily investigates the effects of process parameters, including catalyst type, oxidant concentration, and abrasive particle size in the polishing solution, on the surface quality of SCD within a multi-component reaction system. By integrating single-factor experiments with the AOA, a predictive model was developed to quantify the relationship between these parameters and surface roughness. Key conclusions derived from experimental data analysis are as follows:

(1) The synergistic effect of dual catalysts significantly outperforms single catalysts. In the case of Cu^2+^/Fe^3+^ dual catalysts (SL8), their co-catalysis in the Fenton polishing solution exhibits a synergistic effect: Fe^3+^ activates H_2_O_2_ to generate ·OH radicals, while Cu^2+^ acts as an intermediate to facilitate redox cycling. This collaboration results in a final Sa of 0.112 nm, which is over 60% lower than the surface roughness values obtained with a single Fe^3+^ catalyst (SL6 group, Sa = 0.276 nm) and a single Cu^2+^ catalyst (SL8 group, Sa = 0.294 nm), highlighting the superior efficiency of dual-catalyst systems in promoting material removal and improving surface quality.
(2) The concentration of oxidants significantly influences surface roughness. Under consistent polishing conditions, increasing the oxidant concentration from 15% (Group SL7) to 30% (Group SL5) reduces the Sa from 0.488 nm to 0.276 nm, representing a 43.4% decrease. This result demonstrates a direct correlation between higher oxidant concentrations and enhanced oxidation reactivity, which accelerates chemical etching of the diamond surface and effectively diminishes surface roughness, highlighting the critical role of oxidant dosage in optimizing material removal efficiency and surface quality.
(3) The particle size of abrasive particles significantly influences the surface quality of SCD. Under identical polishing conditions, reducing the abrasive particle size from 500 nm (Group SL7) to 100 nm (Group SL8) optimizes the Sa from 0.154 nm to 0.112 nm. Although no order-of-magnitude change occurs in Sa, the surface quality exhibits fundamental differences: the surface of Group SL7 features periodic scratch-like networks and convex peaks with a scratch depth of 2 nm and peak height of 4 nm, whereas the scratch depth and peak height on the surface of Group SL8 workpieces are reduced by an order of magnitude, demonstrating that smaller abrasive particle sizes significantly minimize mechanical damage and enable a transition from macro-scale scratching to nanoscale surface finishing.
(4) Based on experimental results, the AOA was employed to establish a diamond CMP surface roughness prediction model incorporating three variables, including catalyst type, oxidant concentration and abrasive particle size. The model demonstrates remarkable predictive accuracy, with the root mean square error between predicted and actual values controlled at approximately 0.06, a correlation coefficient reaching 0.998, and relative errors in model validation maintained within 3%, highlighting its high reliability for optimizing polishing process parameters and predicting surface roughness in SCD applications.

Overall, this work not only elucidates the underlying mechanisms of catalytic synergy and surface evolution during SCD polishing but also provides a practical methodology for parameter optimization and surface prediction. The findings hold substantial value for advancing high-precision processing technology of superhard materials, with potential applications in semiconductor, optical, and quantum device manufacturing.

## Figures and Tables

**Table 1:** CMP parameter setting.

Parameters	Types
Disc polishing speed/r/min	60
Diamond rotation speed/r/min	20
Pressure/MPa	1.8
Abrasive type	Al_2_O_3_
Disc throwing type	Glass polishing pad
Abrasive concentration/wt.%	5
Slurry flow rate/mL/min	10
The size of SCD/mm^3^	3 × 3 × 1

**Table 2:** Single-factor experimental parameters and experimental results.

Group Number	H_2_O_2_ Concentration (%)	Abrasive Particle Size (nm)	Fe_2_(SO_4_)_3_ Concentration (mol/L)	CuSO_4_ Concentration (mol/L)	Final Roughness (nm)
SL1	30	500	\	\	1.3
SL2	30	100	\	\	1.2
SL3	30	500	0.2	\	0.6
SL4	30	500	\	0.2	0.7
SL5	30	100	0.2	\	0.3
SL6	30	100	\	0.2	0.4
SL7	30	500	0.2	0.2	0.15
SL8	30	100	0.2	0.2	0.1
SL9	15	500	\	\	1.8
SL10	15	100	\	\	1.6
SL11	15	500	0.2	\	1.2
SL12	15	500	\	0.2	1.4
SL13	15	100	0.2	\	0.5
SL14	15	100	\	0.2	0.6
SL15	15	500	0.2	0.2	0.3
SL16	15	100	0.2	0.2	0.2

**Table 3:** Definition of relevant parameters.

Parameter	Type
*Acc_avg_*	Average acceleration
*Acc_i_^t^*	The acceleration of the *i*-th
α	Density adjustment factor 1
β	Density adjustment factor 2
γ	Volume adjustment factor 1
δ	Volume adjustment factor 2
φ	Resistance adjustment factor 1
ω	Resistance adjustment factor 2
*X_best_*	Optimal surface roughness
*ρ_best_*	Optimal catalyst type
*k_best_*	Optimal oxidant concentration
*V_best_*	Optimal abrasive particle size
*ρ*	Types of catalysts
*V*	Abrasive particle size
*k*	Oxidant concentration
*X*	Polishing quality

**Table 4:** Verification of experimental results.

No.	Measured Value	Predictive Value	Relative Error
S1	1.466	1.502	2.46%
S2	3.936	3.853	−2.11%
S3	0.302	0.308	1.98%
S4	0.125	0.128	2.40%

## Data Availability

The original contributions presented in this study are included in the article. Further inquiries can be directed to the corresponding author.
